# Thoracic Aneurism

**Published:** 1853-10

**Authors:** 


*Thoracic Aneurism.*

In the London Lancet for Sept. we find a report of a case of aneurism, probably of the arch of the aorto, forming a pulsating tumor in the anterior part of the chest, attended with severe cough and dyspnœa. The case was considered at first as one of Phthisis. The progress of the aneurismal sac was very slow, but after several months the development was sufficient for the purpose of making a correct diagnosis. At the end of six months more the tumors were observed to be diminishing in size, until they finally disappeared, although the pulsations still continued, faintly over the locality. The cough was at first increased, but yielded to appropriate treatment. J.

